# Snail transcription factor negatively regulates maspin tumor suppressor in human prostate cancer cells

**DOI:** 10.1186/1471-2407-12-336

**Published:** 2012-08-02

**Authors:** Corey L Neal, Veronica Henderson, Bethany N Smith, Danielle McKeithen, Tisheeka Graham, Baohan T Vo, Valerie A Odero-Marah

**Affiliations:** 1 Center for Cancer Research and Therapeutic Development and Department of Biological Sciences, Clark Atlanta University, Atlanta, GA, 30314, USA; 2 Molecular Urology and Therapeutics Program, Department of Urology and Winship Cancer Institute, Emory University School of Medicine, Atlanta, GA, 30322, USA; 3 The Department of Biological Sciences, Clark Atlanta University, 223 James P Brawley Dr SW Box 1722, Atlanta, GA, 30314, USA

**Keywords:** Snail, Maspin, Prostate cancer

## Abstract

**Background:**

Maspin, a putative tumor suppressor that is down-regulated in breast and prostate cancer, has been associated with decreased cell motility. Snail transcription factor is a zinc finger protein that is increased in breast cancer and is associated with increased tumor motility and invasion by induction of epithelial-mesenchymal transition (EMT). We investigated the molecular mechanisms by which Snail increases tumor motility and invasion utilizing prostate cancer cells.

**Methods:**

Expression levels were analyzed by RT-PCR and western blot analyses. Cell motility and invasion assays were performed, while Snail regulation and binding to maspin promoter was analyzed by luciferase reporter and chromatin immunoprecipitation (ChIP) assays.

**Results:**

Snail protein expression was higher in different prostate cancer cells lines as compared to normal prostate epithelial cells, which correlated inversely with maspin expression. Snail overexpression in 22Rv1 prostate cancer cells inhibited maspin expression and led to increased migration and invasion. Knockdown of Snail in DU145 and C4-2 cancer cells resulted in up-regulation of maspin expression, concomitant with decreased migration. Transfection of Snail into 22Rv1 or LNCaP cells inhibited maspin promoter activity, while stable knockdown of Snail in C4-2 cells increased promoter activity. ChIP analysis showed that Snail is recruited to the maspin promoter in 22Rv1 cells.

**Conclusions:**

Overall, this is the first report showing that Snail can negatively regulate maspin expression by directly repressing maspin promoter activity, leading to increased cell migration and invasion. Therefore, therapeutic targeting of Snail may be useful to re-induce expression of maspin tumor suppressor and prevent prostate cancer tumor progression.

## Background

Snail transcription factor is a zinc finger protein that induces epithelial-mesenchymal transition (EMT) via loss of E-cadherin expression and gain of vimentin expression, leading to increased cell migration, invasion, and tumorigenicity [1-4]. This transcription factor functions as a repressor by having its zinc finger motifs bind to E-boxes along the CDH1 (E-cadherin) promoter thereby repressing transcription (Cano et al., 2000). The expression of Snail and the phenotypical changes associated with EMT have a profound impact of cell movement.

Snail overexpression has been shown in breast cancer and is associated with mammary tumor recurrence [5]. Snail is overexpressed in prostate cancer as well and has been reported to repress Raf kinase inhibitor protein (RKIP) at the transcriptional level in metastatic prostate cancer cell lines [6,7]. Interestingly, androgens (dihydrotestosterone, DHT) has been shown to induce EMT in LNCaP prostate cancer cells by activating Snail, and expression levels of androgen receptor (AR) correlated inversely with androgen-mediated EMT suggesting that low levels of AR was required for the EMT phenotype [8]. Snail can also induce neuroendocrine differentiation in LNCaP cells associated with increased paracrine cell proliferation [9].

Maspin (mammary serine protease inhibitor) is a putative tumor suppressor that is down-regulated during breast and prostate tumor progression [10,11]. It is a serine protease inhibitor that has been shown to regulate urokinase (uPA) and Rac-1 Rho GTPase activities and thus lead to decreased invasion and migration [12-14]. Several mechanisms have been suggested for down-regulation of maspin. Maspin suppression during cancer progression has been shown to be mediated by promoter methylation in several cancers including breast cancer [15,16]. Transcription factors like mutant p53 and AR have also been shown to bind to maspin promoter and mediate its inhibition in prostate cancer [13,17,18]. The maspin promoter contains a negative regulatory hormone response element (HRE) that can be bound by AR leading to inhibition of maspin promoter activity [13,18]. Although the effect of maspin has been studied in several cancers, there is no report that correlates the expression of maspin with Snail.

Previously, we have shown that Snail promotes EMT in ARCaP and LNCaP cells associated with increased cell migration [9,19]. In this study, we utilized normal and prostate cancer cell lines to show that Snail overexpression in cancer correlates inversely with maspin down-regulation. We showed that Snail may inhibit maspin protein expression by directly binding maspin promoter, resulting in repression of maspin promoter activity. This may explain one of the many mechanisms by which maspin is lost during tumor progression and opens up novel therapeutic avenues by which we could essentially target Snail to re-express maspin resulting in a halt to tumor progression in prostate cancer.

## Methods

### Reagents and antibodies

RPMI medium and penicillin/streptomycin were purchased from VWR Int., West Chester, PA. The protease inhibitor cocktail was from Roche Molecular Biochemicals, Indianapolis, IN. Mouse monoclonal anti-human maspin antibody was from BD Transduction Laboratories, Lexington, KY. G418 and anti-human actin antibodies were from Sigma-Aldrich, Inc., St Louis, MO. Rat monoclonal anti-human Snail antibody and HRP-conjugated goat anti-rat antibody were from Cell Signaling Technology, Inc., Danvers, MA. HRP-conjugated sheep anti-mouse, sheep anti-rabbit and the Enhanced chemiluminescence (ECL) detection reagent were purchased from Amersham Biosciences, Buckingham, England. Fetal bovine serum (FBS) and Charcoal/dextran treated FBS (DCC-FBS) were from Hyclone, South Logan, UT. The pGL3-basic vector, β-galactosidase cDNA, Sac I and Bgl II restriction enzymes were purchased from Promega, Madison, WI. The Snail cDNA construct was kindly provided by Dr Mien-Chie Hung, University of Texas, Houston, TX. Control and Snail siRNA constructs were from Dharmacon, Lafayette, Co. The full length maspin promoter in pCR2.1TOPO vector were a kind gift from Dr Zhila Khalkhali-Ellis, Children’s Memorial Research Center, Chicago, Il. Lipofectamine 2000 was from Invitrogen, Carlsbad, CA. The EZ-ChIP kit was purchased from Millipore Inc., Billerica, MA.

### Cell culture

Normal prostate epithelial PrEC cells (Clonetics-Biowhittaker) were cultured in PrEMB medium. The human prostate cancer cell lines, LNCaP, 22Rv1 and DU145, were obtained from ATCC, Manassas, VA. The LNCaP, C4-2 human prostate cancer progression model was established as described previously [20], while generation of C4-2 cells with stable Snail knockdown has been reported previously [21]. Cells were grown in RPMI medium supplemented with 5% fetal bovine serum and 1X penicillin-streptomycin, at 37°C with 5% CO_2_ in a humidified incubator.

### Western blot analysis

Confluent cells were lysed in a modified RIPA buffer (50 mM Tris, pH 8.0, 150 mM NaCl, 0.02% NaN3, 0.1% SDS, 1% NP-40, 0.5% sodium deoxycholate) containing 1.5X protease inhibitor cocktail, 1 mM phenylmethylsufonyl fluoride, and 1 mM sodium orthovanadate. The cell lysates were centrifuged, and supernatants collected and quantified using a micro BCA assay. 25–30 μg of cell lysate was resolved on a 4-12% SDS PAGE, followed by transblotting onto nitrocellulose membrane (Schleicher & Schuell, Keene, NH). The membranes were blocked in TBS-TB (TBS with 0.05% Tween-20, 0.05% BSA) containing 5% milk, and subsequently incubated with diluted antibody in blocking buffer. After washing, the membranes were incubated in peroxidase-conjugated sheep anti-mouse, sheep anti-rabbit, or goat anti-rat IgG, washed, and visualized using ECL reagent. The membranes were stripped using stripping buffer (Pierce Biotechnology, Inc., Rockford, IL) prior to re-probing with a different antibody.

### Transfection assay

Stable transfection of Snail cDNA was performed in 22Rv1 cells utilizing Lipofectamine 2000. The Snail cDNA is the constitutively active construct (6SA) that was previously utilized to induce EMT in MCF7 breast cancer cells [22]. Briefly, 1.6 μg Snail cDNA or empty vector (Neo) was transfected into cells cultured in 12 well dishes at 90% confluency as per manufacturer’s instructions. Stable clones were selected using 800 μg/ml G418, isolated, and maintained in 400 μg/ml G418. Snail expression was verified in the clones by Western blot analysis.

### RNA Isolation and RT-PCR

Total RNA was isolated from cells using the Qiagen kit as per manufacturer’s instructions, and 1 μg reverse transcribed with oligo-dT using MMLV-reverse transcriptase (Invitrogen), to generate cDNA. PCR analyses were subsequently performed with 2 μl of cDNA utilizing the primers and conditions as follows: Snail primers were 5′-GCTCGAAAGGCCTTCAACTGCAAA-3′ and 5′-AGGCAGAGGACACAGAACCAGAAA-3′, Maspin primers were 5′-CTGACAACAGTGTGAACGAC-3′ and 5′-CAAGCCTTGGGATCAATCATCT-3′, and GAPDH primers were 5′-GAAGGTGAAGGTTCGGAGTC-3′ and 5′-GAAGATGGTGATGGGATTTC-3′. The PCR conditions for Snail and GAPDH were 94°C, 2 min, 29 cycles of 94°C, 30 s; 55°C, 30 s; 72°C, 2 min, and 72°C, 7 min final extension, while for maspin it was 95° 5 min, 35 cycles of 94° 1 min, 56° 30 secs, 72° 1 min, and 72° 5 min final extension.

### siRNA treatment

DU145 or 22Rv1 Snail-transfected cells at 70% confluency were transfected with 200 nM control or Snail smartpool siRNA (Dharmacon) using Dharmafect I reagent, as per manufacturer’s instructions, for 72 h prior to isolation of protein for western blot analysis.

### In vitro cell migration and invasion assay

We utilized Costar 24-well plates containing a polycarbonate filter insert with an 8-μ pore size, coated with collagen I on the outside for migration or matrigel on the inside for invasion assays. 50,000 cells were plated in the upper chamber containing 0.1% fetal bovine serum (FBS) while the lower chamber contained 10% FBS. 24 h later, cells that had migrated to the bottom of the insert was fixed, stained, and either counted to obtain the relative migration or the stain solubilized with Sorenson solution and OD measured at 490 nm to obtain relative migration.

### Maspin promoter luciferase-reporter assay

The full length maspin promoter [18] in pCR2.1TOPO vector was double-digested with Sac I and Bgl II, ligated into pGL3-basic vector and DNA sequences of the constructs confirmed by DNA sequencing (Morehouse School of Medicine DNA Facility). 22Rv1 cells overexpressing Snail or C4-2 cells with stable Snail knockdown were plated at 6 x 10^5^ cells/well in 6-well dishes in hormone-depleted media. Cultures were transfected with 3 μg of DNA from the full-length maspin promoter reporter plasmids and an internal renilla luciferase plasmid for transfection efficiency, using Lipofectamine 2000. After 48 h, the cells were harvested in reporter lysis buffer (Promega), and supernatant(s) were used to determine luciferase activity using the Dual-Glo Luciferase Assay System (Promega) according to the manufacturer’s instruction. The results were expressed as the increased induction (or suppression) of the reporter plasmid after normalization against the internal control plasmid.

### ChIP Assay

22RV1 cells either stably expressing Neo vector control (Neo10 clone) or Snail cDNA (Snail30 clone) were used for ChIP assay using the EZ-ChIP kit. The cells were cross-linked with formaldehyde for 10 min at 37°C with mild shaking, washed in ice cold PBS, unreacted formaldehyde was quenched with glycine, then washed with PBS and resuspended in SDS buffer. Samples were sonicated to approximately 600 bps with Sonicator (Misonix Sonicator S-3000), diluted in dilution buffer with inhibitors and precleared with agarose G beads. The supernatant was used directly in immunoprecipitation with anti-Snail, IgG (for negative control) or RNA polymerase II (for positive control). The immunocomplexes were mixed with 120 μl of DNA coated agarose G beads followed by incubation overnight. Pellets were washed in a low salt wash buffer (x1), high salt wash buffer (x1), LiCl wash buffer (x1) and TE buffer (x2). This was followed by adding 200 μl of elution buffer to elute the protein/DNA complex and cross-linking was reversed by adding 5 M NaCl with incubation overnight. The protein was then digested by addition of 1 μl proteinase K to each sample followed by incubation for 2 hrs. DNA was purified by washing with elution buffer and centrifugation and then subsequently processed by PCR.

### Quantitative Real-Time PCR (QRT-PCR)

2 μl of the DNA eluates from the ChIP assay were added into a 96 well QPCR plate for each corresponding sample. Subsequently a master mix was made using maspin promoter primers (catalog number GPH1006313(−)01A, from SA Biosciences, Frederick, MD), and the RT2 qPCR mastermix reagent (catalog number PA-011, from SA Biosciences) according to manufacterer’s instructions. QRT-PCR was then done using an I-cycler (Bio-Rad) to quantitate transcript levels by the SYBR Green method. Cycle threshold differences were then determined using an I-cycler (Bio-Rad) relative to input chromatin (chromatin initially used for the immunoprecipation). Fold changes in transcript levels of maspin gene were then calculated in samples immunoprecipitated with either RNA polymerase II (positive control), mouse IgG (negative control), or Snail antibody. The results were graphed and the standard error determined. Samples were also resolved on an agarose gel. As another control PCR was performed with primers to maspin intronic region. The primer sequence was Forward: 5′- AGGAGCCAGTCAGCATAGGA- 3′ and Reverse: 5′- TTTGGCTGCAAACACCTACA- 3′.

## Results

### Snail overexpression negatively correlates with maspin expression

**Figure 1:**
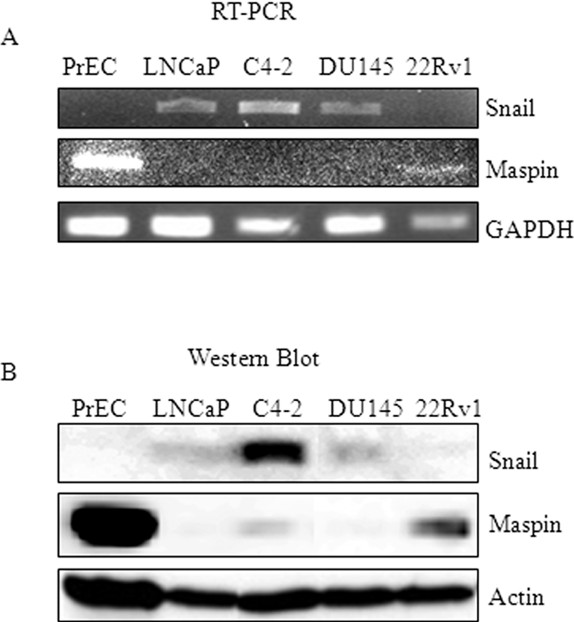
**Snail expression correlates inversely with maspin expression in normal and prostate cancer cell lines.** Normal immortalized epithelial cells (PrEC), the LNCaP prostate cancer progression model (LNCaP, C4-2), DU145 and 22Rv1 cells were utilized to analyze Snail and maspin levels by **(A)** PCR and **(B)** Western blot analyses. GAPDH and actin were utilized as loading controls for PCR and Western blot analysis, respectively. All experiments were performed at least 3 times.

We examined the expression of Snail transcription factor in normal prostate epithelial cells and different prostate cancer cell lines by RT-PCR and Western blot analysis. The normal prostate epithelial cells (PrEC) failed to express detectable levels of Snail, while Snail was readily detectable in the prostate cancer cell lines LNCaP, the LNCaP derivative cell line C4-2, DU145, and not detectable in 22Rv1 cells (Figure 1A, B). Conversely, PReC normal epithelial cells expressed high levels of maspin as compared to the prostate cancer cell lines (Figure 1A, B). This demonstrates that Snail expression is inversely correlated with maspin expression in normal epithelial prostate cells and prostate cancer cell lines.

### Overexpression of Snail in 22Rv1 prostate cancer cells leads to decreased expression of maspin and increased migration/invasion

**Figure 2:**
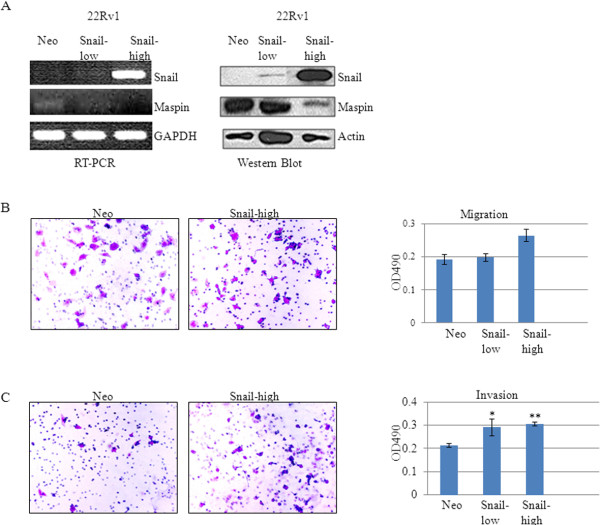
**Snail overexpression in 22Rv1 cells leads to decreased maspin expression, and increased migratory and invasive potential.** 22Rv1 prostate cancer cells were stably transfected with Snail cDNA or empty vector control (Neo) using lipofectamine 2000. **(A)** A representative Snail clone (Snail-high) that expressed the highest levels of Snail, as assayed by PCR and Western blot analysis, also expressed the least amount of maspin, when compared to Neo control and the low-expressing Snail clone (Snail-low). **(B)** Snail overexpression in 22Rv1 cells was accompanied by increased migration on collagen and **(C)** increased invasion on matrigel in the Snail-high clone. Results are representative of three independent experiments. Data represent mean ± SD (* p < 0.05, **p < 0.01).

Since we had observed an inverse relation between Snail and maspin, we sought to investigate whether Snail could regulate maspin expression. We decided to utilize androgen-dependent 22Rv1 cells to represent a prostate cancer cell model that expresses undetectable levels of Snail in order to overexpress Snail and subsequently examine maspin expression. We utilized lipofectamine 2000 to overexpress Snail cDNA or empty vector control (Neo) in 22Rv1 prostate cancer cells. Stable clones were selected with G418 and tested for expression of Snail and maspin by RT-PCR and Western blot analysis. 22Rv1 Snail clone high demonstrated the highest levels of Snail that corresponded with the lowest levels of maspin when compared to 22Rv1 Neo control or Snail low clone that expressed low levels of Snail by RT-PCR and Western blot (Figure 2A). 22Rv1 Snail high clone, which displayed the highest levels of Snail, also displayed increased migration (p = 0.06) and invasion (**p < 0.01) as compared to 22Rv1 Neo control (Figure 2B, C). These results suggest that Snail overexpression can result in maspin inhibition as well as increased migration and invasion in 22Rv1 prostate cancer cells.

### Knockdown of Snail expression can reinduce maspin expression

**Figure 3:**
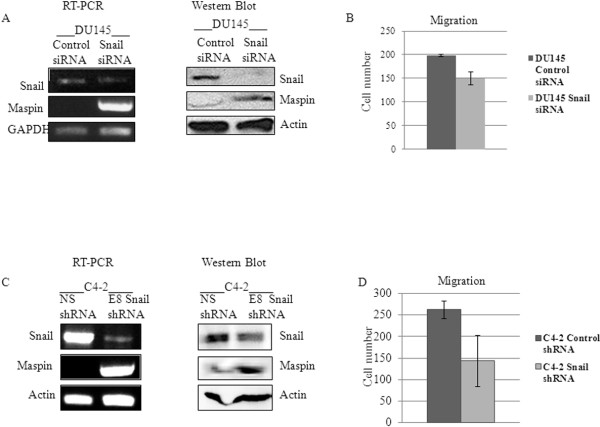
**Downregulation of Snail in DU145 and C4-2 cells is associated with increased maspin expression and decrease in cell migratory potential.(A)** DU145 cells were transiently transfected with control or Snail siRNA for 3 days followed by PCR and Western blot analysis for Snail and maspin expression. **(B)** 5X10^4^ DU145 cells that had been treated with control or Snail siRNA was plated in a Boyden chamber and tested for migration on collagen. There was a decrease in migration (mean ± SD, p = 0.071). **(C)** C4-2 cells stably transduced with non-silencing control (NS) or Snail shRNA lentiviral vectors (E8) displayed decreased Snail and increased maspin expression by PCR and Western blot analysis. **(D)** 5X10^4^ C4-2 control- or Snail shRNA-expressing cells were tested for migration on collagen using a Boyden chamber. There was a decrease in migration (mean ± SD, p = 0.072) Results are representative of triplicate experiments performed independently.

Next we examined whether inhibition of Snail in prostate cancer cells could lead to maspin reexpression. We utilized AR-negative DU145 cells and AR-positive C4-2 cells to check whether AR was required for regulation of maspin by Snail. We also utilized these cell lines because they represent androgen-independent, aggressive cell lines that express higher levels of Snail. DU145 cells transfected with Snail or control siRNA for 3 days were examined for Snail and maspin expression as well as migratory potential on collagen using a Boyden chamber assay. The data showed that Snail knockdown did result in decreased Snail mRNA and protein expression, increased maspin expression and decreased cell migration (p = 0.071) when compared to control siRNA treatment (Figure 3A, B). Similarly, stable knockdown of Snail in C4-2 cells using shRNA (C4-2 E8) resulted in decreased expression of Snail, increased expression of maspin, and decreased cell migration (p = 0.072) when compared to control non silencing shRNA (C4-2 NS) expressing cells (Figure 3C, D). Therefore, Snail knockdown may alleviate maspin inhibition in AR-negative and –positive prostate cancer cell lines.

### Snail negatively regulates activity of maspin promoter

**Figure 4:**
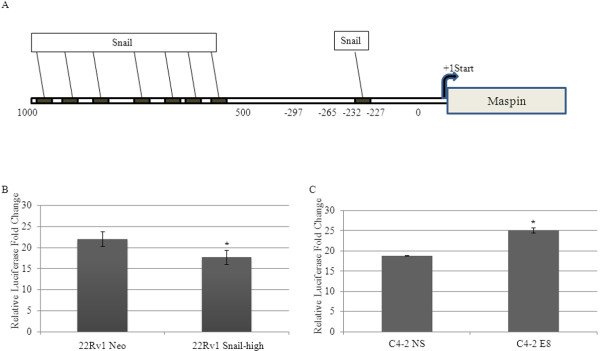
**Snail can negatively regulate maspin promoter activity.(A)** Analysis of maspin promoter region with ConSite software revealed 8 E-boxes. 22Rv1 cell lines stably overexpressing Snail (Snail-high clone) or vector control (Neo) or C4-2 cells with stable Snail knockdown (C4-2 E8) or non-silencing control (C4-2 NS) were plated at 6 x 10^5^ cells/well in 6-well dishes overnight in hormone-depleted media. Cells were subsequently transfected with 3 μg of DNA from the full-length maspin promoter reporter plasmid and an internal control plasmid (renilla luciferase for transfection efficiency) using Lipofectamine 2000. After 48 h, analysis of luciferase activity relative to renalla luciferase activity showed that **(B)** maspin promoter activity was decreased in 22Rv1 cells overexpressing Snail and **(C)** maspin promoter activity was increased in C4-2 cells with stable Snail knockdown. The experiments were performed in triplicate at least three times independently. Bars, SD *, P < 0.05, Student’s t test compared with control.

We sought to examine the molecular mechanisms by which Snail may be inhibiting maspin expression. We found 8 E-box elements (consensus sequence that Snail binds to which is CAGGTG or CANNTG) within the maspin promoter, 1000 bps upstream of the start site, using ConSite software (Figure 4A). Therefore, we hypothesized that Snail may regulate maspin at the promoter level. We obtained full length maspin promoter and ligated it to the luciferase vector. We utilized 22Rv1 and LNCaP cells with stable Snail overexpression or C4-2 cells with stable endogenous Snail knockdown to examine maspin promoter activity by transiently transfecting full length maspin promoter (Maspin-Luc) plus renalla luciferase vector as an internal control, for 48 h. We also utilized parental LNCaP or 22Rv1 prostate cancer cells transiently co-transfected with Snail or Neo cDNA and Maspin-luc for 48 h and β-galactosidase (β-gal) as an internal control. Subsequently, luciferase activity was measured and normalized to renalla luciferase or β-gal. Transient or stable Snail transfection led to significantly decreased maspin promoter activity as compared to Neo control in both 22Rv1 and LNCaP cells (Figure 4B, Additional file 1: Figure S1). Conversely, stable knockdown of Snail in C4-2 cells (C4-2 E8) increased maspin promoter activity as compared to C4-2 NS non-silencing control (Figure 4C). These results suggest that Snail can negatively regulate maspin promoter activity.

### Snail transcription factor binds to maspin promoter

**Figure 5:**
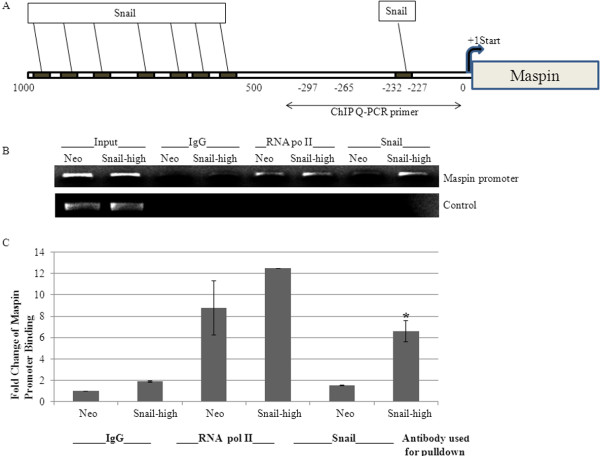
**Snail binds to the maspin promoter.** 22Rv1 cell lines stably overexpressing Snail (Snail-high clone) or vector control (Neo) were utilized to perform ChIP analysis. **(A)** The maspin promoter region is shown with the putative E-boxes; the ChIP Q-PCR primer recognized the first E-box upstream of the start site. **(B)** Chromatin was immunoprecipitated with mouse IgG as a negative control, anti-RNA polymerase II antibody as a positive control or anti-Snail antibody. Real-time PCR was subsequently performed with primers that recognize the maspin promoter within the first E-box upstream of the start site. The samples were run on an agarose gel and input included as control for loading. ChIP PCR was also performed with primers to maspin intron as another negative control. **(C)** The results of the real-time PCR were plotted as fold change of binding to maspin promoter. The experiments were performed in triplicate at least three times independently. Bars, SD *, P < 0.05, Student’s t test compared with 22Rv1 Neo control.

Because we had shown that Snail can negatively regulate maspin promoter activity, we investigated whether Snail can physically bind to the maspin promoter. A ChIP assay was performed using 22Rv1 Neo or Snail-transfected cells to immunoprecipitate Snail from chromatin and perform real-time PCR with maspin promoter primers that spanned the first E-box upstream of the start site (Figure 5A). Mouse IgG was utilized as a negative control while anti-RNA Polymerase II antibody was utilized as a positive control. ChIP-PCR was also performed with maspin intron primers as another control. The data revealed Snail binding to maspin promoter about 6-fold greater in 22Rv1 Snail cells as compared to Neo control cells (Figure 5B, C). Therefore, Snail overexpression may lead to maspin repression in part through binding of Snail to maspin promoter.

## Discussion

Our research focused on studying the mechanism(s) by which Snail transcription factor may contribute to cancer progression in prostate cancer. One of the ways by which Snail can lead to cancer progression is through induction of epithelial-mesenchymal transition (EMT), which involves the loss of epithelial markers such as E-cadherin, and acquisition of mesenchymal markers such as vimentin [2]. Snail can negatively regulate a number of tumor suppressors including E-cadherin, claudins, and occludin, by binding to E-boxes in the promoter region [2,23,24]. This communication studied the relationship between Snail and maspin tumor suppressor, to discover a new mechanism by which maspin may be downregulated during prostate tumor progression.

Maspin tumor suppressor has been shown to be downregulated in breast and gastric cancer through promoter methylation [15,25,26]. Maspin expression is also lost with prostate tumor progression, through inactivation of a positive Ets response element and activation of a negative HRE response element recognized by AR [18]. Recently, interleukin-6 (IL-6) signaling has been shown to downregulate maspin expression [27].

The present study correlates Snail expression with prostate cancer, as Snail protein was absent in normal immortalized prostate epithelial cells (PrEC), however it was then expressed in our LNCaP progression model (LNCaP, C4-2), DU145 prostate cancer cell lines, though undetectable in 22Rv1 cells. Conversely, maspin expression was high in PrEC and low in the prostate cancer cell lines. The inverse relationship between Snail and maspin led us to investigate whether Snail may be negatively regulating maspin expression. Indeed, we found that when Snail is overexpressed in 22Rv1 cells, maspin expression was decreased, while migratory and invasive potential increased. Conversely, when Snail expression was inhibited with siRNA or shRNA in DU145 or C4-2 cells, respectively, maspin expression increased, while migratory potential decreased. This study reports evidence for the first time, that Snail oncogene can negatively regulate maspin tumor suppressor. Since maspin is silenced epigenetically in some cancers, studies aim at preventing tumor progression by reinducing maspin expression with methylation inhibitors such as 5- aza-2 ′-deoxycytidine and histone deacetylase inhibitors [28-30]. These are general inhibitors that would lead to non-specific demethylation. We provide a novel mechanism by which therapeutic targeting of Snail in the future, may prevent tumor cell migration by reinducing maspin expression.

We have also utilized LNCaP and 22Rv1 cells transiently or stably transfected with Snail to show that Snail does significantly reduce maspin promoter activity, while knockdown of endogenous Snail in C4-2 cells increased maspin promoter activity. To elucidate the mechanism, we have found 8 E-boxes within the maspin promoter and showed that Snail directly binds to the maspin promoter in 22Rv1 cells. Our data suggest that Snail may repress maspin independently of AR since knockdown of Snail resulted in decreased maspin expression in both AR-negative DU145 and AR-positive C4-2 cells. It is also possible that Snail may negatively regulate maspin by recruiting histone deacteylases (HDACs). Although Snail has been shown to directly bind to the E-cadherin promoter, it can also repress E-cadherin epigenetically by recruiting a corepressor, Ajuba LIM domain protein resulting in histone modifications and promoter methylation [31,32]. It was reported that receptor activator of NF-kappa B ligand (RANKL) signaling to Ikappa B kinase alpha (IKKalpha) represses maspin expression in prostate epithelial cells, associated with nuclear translocation of IKKalpha [33]. We have previously shown that Snail can induce the expression of RANKL [19], so it is possible that Snail may be repressing maspin through the RANKL-IKKalpha pathway. Alternatively, p53 has been shown to bind to maspin promoter leading to activation of its transcription [34,35], while Snail interacts directly with the DNA binding domain of p53 diminishing its tumor suppressive function [36], therefore, it seems plausible that Snail may inhibit maspin via p53 pathway. Thus although we report one step in which Snail directly binds to maspin promoter to inhibit its promoter activity and expression, this does not exclude other possibilities by which Snail may negatively regulate maspin.

## Conclusions

Collectively, our results indicate for the first time that Snail can negatively regulate maspin through direct promoter repression resulting in increased migration and invasion in prostate cancer cells. This study reveals a novel mechanism of how Snail may function and show the importance of therapeutic targeting of Snail signaling in future.

## Competing interests

The authors declare that they have no competing interests.

## Authors’ contributions

VOM designed the research studies. CLN, VH, BS, DMK, TG, and BTV carried out the experiments; VOM and CLN analyzed and interpreted the data; CLN and VOM wrote the draft of the manuscript. All authors read and approved of the final manuscript.

## Pre-publication history

The pre-publication history for this paper can be accessed here:

http://www.biomedcentral.com/1471-2407/12/336/prepub

## Supplementary Material

Additional file 1 **Snail overexpression represses maspin promoter activity in LNCaP and 22Rv1 cells.** We transiently co-transfected LNCaP or 22Rv1 prostate cancer cells with Snail or Neo cDNA and full length maspin promoter (Maspin-luc) for 48 h. We also utilized LNCaP cells stably overexpressing Snail as shown by PCR analysis (Snail-medium and Snail-high clones) as compared to the Neo control (LNCaP Neo) as shown in Additional file 1: Figure S1C, and used the representative Snail-high clone to analyze maspin promoter activity. As an internal control, all cells were transfected with β-galactosidase (β-gal) for the transient transfections and renilla luciferase for the stable transfection. Subsequently, luciferase activity was measured and normalized to β-gal or renilla luciferase. Snail transfection led to significantly decreased maspin promoter activity as compared to Neo transfection in both LNCaP and 22Rv1 cells (Additional file 1: Figure S1). These results suggest that Snail can negatively regulate maspin promoter activity. Click here for file
