# Supplementary material for: Snail transcription factor negatively regulates maspin tumor suppressor in human prostate cancer cells
Source: BMC Cancer. 2012 Aug 2;12:336. doi: 10.1186/1471-2407-12-336 (PMC3437215; doi:10.1186/1471-2407-12-336)
Supplement: Additional file 1 — Snail overexpression represses maspin promoter activity in LNCaP and 22Rv1 cells. We transiently co-transfected LNCaP or 22Rv1 prostate cancer cells with Snail or Neo cDNA and full length maspin promoter (Maspin-luc) for 48 h. We also utilized LNCaP cells stably overexpressing Snail as shown by PCR analysis (Snail-medium and Snail-high clones) as compared to the Neo control (LNCaP Neo) as shown in Additional file 1: Figure S1C, and used the representative Snail-high clone to analyze maspin promoter activity. As an internal control, all cells were transfected with β-galactosidase (β-gal) for the transient transfections and renilla luciferase for the stable transfection. Subsequently, luciferase activity was measured and normalized to β-gal or renilla luciferase. Snail transfection led to significantly decreased maspin promoter activity as compared to Neo transfection in both LNCaP and 22Rv1 cells (Additional file 1: Figure S1). These results suggest that Snail can negatively regulate maspin promoter activity. [file 1471-2407-12-336-S1.doc]

**Supplementary Methods**

**Transfection assay**

Transient transfection of Snail cDNA was performed in LNCaP and 22Rv1 cells utilizing Lipofectamine 2000 for 48 h. Stable transfection of Snail cDNA was performed in LNCaP cells utilizing Lipofectamine 2000. The Snail cDNA is the constitutively active construct (6SA) that was previously utilized to induce EMT in MCF7 breast cancer cells (Zhou et al., 2004). Briefly, 1.6 μg Snail cDNA or empty vector (Neo) was transfected into cells cultured in 12 well dishes at 90% confluency as per manufacterer’s instructions. Stable clones were selected using 800 μg/ml G418, isolated, and maintained in 400 μg/ml G418. Snail expression was verified in the clones by PCR analysis.

**RNA Isolation and RT-PCR**

Total RNA was isolated from cells using the Qiagen kit as per manufacturer’s instructions, and 1 μg reverse transcribed with oligo-dT using MMLV-reverse transcriptase (Invitrogen), to generate cDNA. PCR analyses were subsequently performed with 2 μl of cDNA utilizing the primers and conditions as follows: Snail primers were 5’-GCTCGAAAGGCCTTCAACTGCAAA-3’

and 5’-AGGCAGAGGACACAGAACCAGAAA-3’,

Maspin primers were 5’-CTGACAACAGTGTGAACGAC-3’ and 5’-CAAGCCTTGGGATCAATCATCT-3’, and GAPDH primers were 5’-GAAGGTGAAGGTTCGGAGTC-3’ and 5’-GAAGATGGTGATGGGATTTC-3’. The PCR conditions for Snail and GAPDH were 94^o^C, 2 min, 29 cycles of 94^o^C, 30 s; 55^o^C, 30 s; 72^o^C, 2 min, and 72^o^C, 7 min final extension, while for maspin it was 95° 5 min, 35 cycles of 94° 1 min, 56° 30 secs, 72° 1 min, and 72° 5 min final extension.

**Maspin promoter luciferase-reporter assay**

LNCaP or 22Rv1 cells were plated at 6 x 10^5^ cells/well in 6-well dishes in hormone-depleted media and transiently transfected with 3 µg of DNA from the full-length maspin promoter reporter plasmid (Maspin-luc) plus empty vector Neo or Snail cDNA using Lipofectamine 2000, for 48 h. β-galactosidase (β-gal) was also transfected as an internal control. After 48 h, the cells were harvested in reporter lysis buffer (Promega), and supernatant(s) were used to determine luciferase activity using the Luciferase Assay System (Promega) according to the manufacturer’s instruction. The results were expressed as the increased induction (or suppression) of the reporter plasmid after normalization against the internal control plasmid. LNCaP cells were transfected with empty vector (Neo) or Snail cDNA and selection of stable clones performed with 800 mg/ml G418. Stable clones were maintained in 400 mg/ml G418. LNCaP stable overexpression cell lines were co-transfected with Maspin-luc and renilla luciferase as the internal control for 48 h. Luciferase assay was measured using the Dual-Glo luciferase assay (Promega) and results normalized to the internal control.

**Supplementary Figure 1**

Snail overexpression represses maspin promoter activity in LNCaP and 22Rv1 cells. We transiently co-transfected LNCaP or 22Rv1 prostate cancer cells with Snail or Neo cDNA and full length maspin promoter (Maspin-luc) for 48 h. We also utilized LNCaP cells stably overexpressing Snail as shown by PCR analysis (Snail-medium and Snail-high clones) as compared to the Neo control (LNCaP Neo) as shown in Supplemental Fig. 1C, and used the representative Snail-high clone to analyze maspin promoter activity. As an internal control, all cells were transfected with β-galactosidase (β-gal) for the transient transfections and renilla luciferase for the stable transfection. Subsequently, luciferase activity was measured and normalized to β-gal or renilla luciferase. Snail transfection led to significantly decreased maspin promoter activity as compared to Neo transfection in both LNCaP and 22Rv1 cells (Fig. S1). These results suggest that Snail can negatively regulate maspin promoter activity.


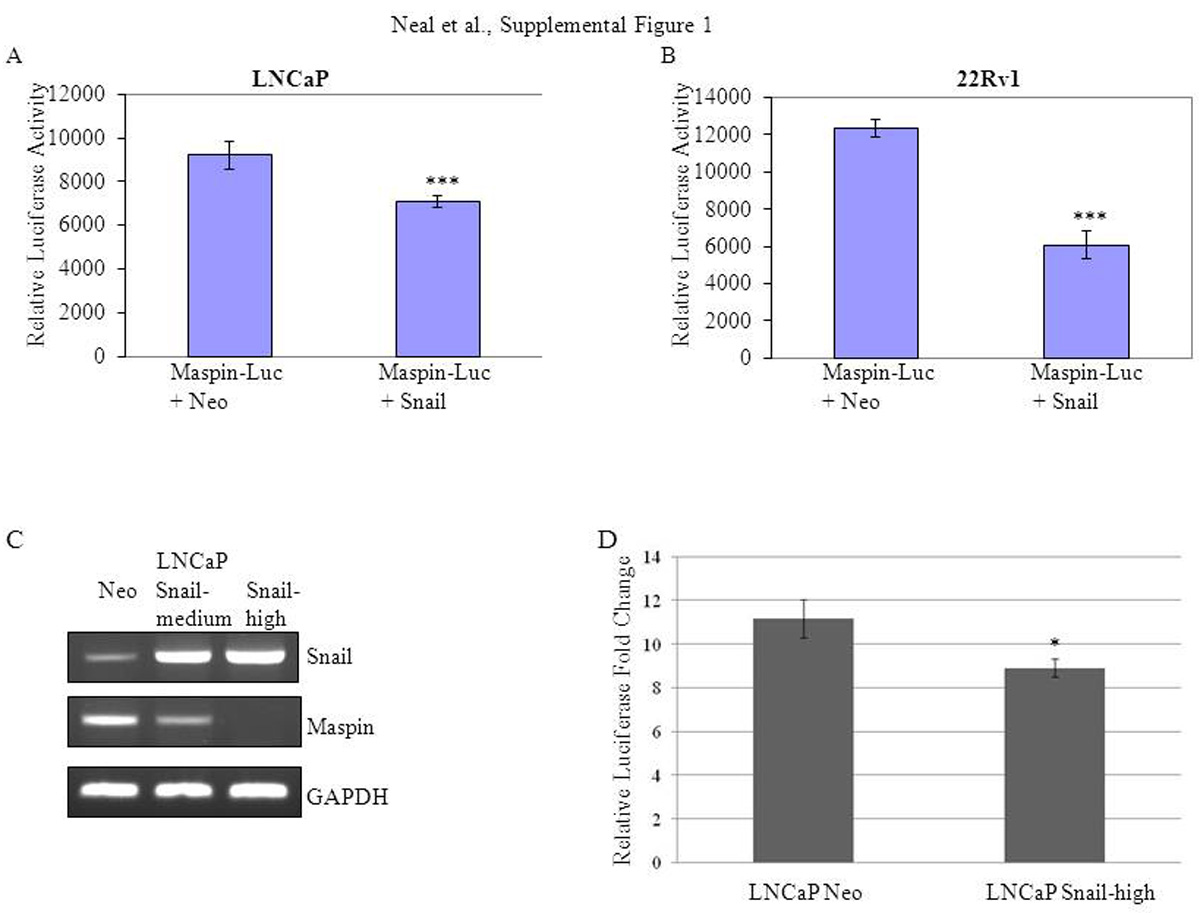


**Supplementary Figure Legends**

**Figure S1**

**Snail can negatively regulate maspin promoter activity.** Full length maspin promoter (Maspin-luc) was co-transfected with β-galactosidase (β-gal) and Snail cDNA vector into (A) LNCaP or (B) 22Rv1 cells for 48 h. Subsequently, cells were lysed and analyzed for luciferase activity relative to β-gal control. (C) LNCaP cells were stably transfected with empty vector (Neo) or Snail cDNA using Lipofectamine 2000 and stable clones selected with G418. LNCaP Snail-medium and -high clones expressed more Snail and less maspin by PCR analysis as compared to LNCaP Neo clone. (D) LNCaP Neo or LNCaP Snail-high cells were transfected with Maspin-luc plus renilla luciferase internal control for 48 h and luciferase activity assayed and normalized to internal control. The experiments were performed in triplicate at least twice independently. Bars, SD *, P < 0.05; ***, P < 0.001, Student’s t test compared with Neo.
